# Oncology Information System: A Qualitative Study to Identify Cancer Patient Care Workflows

**DOI:** 10.25122/jml-2019-0169

**Published:** 2020

**Authors:** Azadeh Yazdanian

**Affiliations:** 1. Department of Medical Record and Health Information Technology, School of Allied Medical Sciences,Mazandaran University of Medical Sciences, Sari, Iran

**Keywords:** Neoplasm, oncology information system, workflows, qualitative study, hospital

## Abstract

Oncology information systems provide solutions for managing the information of cancer patients and enable monitoring of different aspects of cancer patient care. Since the use of oncology information systems enhances the quality of care, improves documentation, optimizes resource allocation, and increases the cost-effectiveness of care services, attention to these systems’ performance and their adaptation to workflows seems necessary. The purpose of this study was to identify cancer patient care workflows to design an oncology information system for Iran.

This study employed a qualitative design and was conducted in 2019. Semi-structured interviews were conducted with 25 experts to determine their views on identifying workflows for cancer patients’ care. The participants were clinical and non-clinical staff at six university hospitals equipped with oncology wards. The method of data analysis was framework analysis.

The cancer patient care workflows consisted of two categories, including cancer diagnosis workflows and cancer treatment workflows. Cancer diagnosis workflows fall into three subcategories, i.e., the patient’s referral to the clinic, an examination of the patient’s condition, and pathology workflows. On the other hand, cancer treatment workflows are divided into various treatments offered to cancer patients and workflows in the chemotherapy and radiotherapy wards.

Given the variety of services and the complexity of caring for cancer patients as well as the involvement of various specialists in the process of care, identifying and optimizing workflows in the oncology information system reduces errors, enhances data accuracy, eliminates unnecessary steps, and ultimately improve the service delivery to cancer patients.

## Introduction

By controlling infectious diseases and increasing life expectancy in the world, non-communicable and chronic diseases such as cancers are the most important causes of mortality [1]. According to the World Health Organization, about 8.2 million people die each year from cancer. Given that early and proper diagnosis and effective treatment can help increase the survival rate of cancer patients, the development of different cancer treatment methods has increased survival in most developed countries in recent decades [2].

Advances in the development of various diagnostic and treatment methods for cancers depend on the collection and analysis of relevant data. Therefore, given the growing volume of clinical data on cancer patients, designing and deploying clinical information systems in the field of cancer to manage relevant data seems inevitable [3]. One of the cancer data management systems used in hospitals is the Oncology Information System. The system supports the provision of integrated care and long-term treatment of cancer patients by collecting data on different stages of cancer patient referral (prevention, screening, diagnosis, treatment, palliative care, and end-of-life care) [4].

Currently, the variety of imaging modalities plays an important role in the detection, diagnosis, grading, and monitoring of cancers [5, 6]. Cancer care has also become very complicated due to the emergence of new drugs, new regimens, and a variety of poisonings and treatments. Given that various professionals including oncologists, pathologists, radiologists, pharmacists, and laboratory technicians are involved in clinical processes of cancer care, identifying and optimizing workflows in clinical information systems in the field of cancer reduces errors, increases data accuracy, enhances efficiency through automating activities, eliminate unnecessary steps, improves management through standard operating practices, and ultimately enhances patient service delivery [7].

Numerous studies have been conducted on cancer patient care workflows, some of which are reviewed here. Yang conducted a study to create a mockup to predict the workflows in the radiation therapy ward and implement them on a tablet. First, the paper forms were examined, then the users and scenarios were considered, and finally, the user interfaces were designed. The results of interviews with users showed that the use of electronic protocols was a good way to support their workflows [2].

Wagner conducted a study to define integrated clinical documentation to support workflows for all types of cancers at Erlangen University Hospital. In this study, diagnostic and therapeutic strategies for 13 types of cancers were collected using a combined approach, including document analysis, workflow analysis, expert interviews, workflow modeling, and feedback cycles in 14 steps. Then three main categories of diagnostic and therapeutic strategies were identified, and for each, an integrated documentation approach could be applied to support workflows [8]. Liu and Wen conducted a study to develop and evaluate an information system for chemotherapy care. They divided the chemotherapy process into four stages, each requiring the system registration and confirmation. These stages included prescribing medications by an oncologist, transcribing by a nurse, distributing medication by a pharmacist, and administration and injection by a nurse. After the design, the system was fully implemented and evaluated six months later.

The evaluation results showed that using the barcode-based drug management system resulted in the reduction of medication errors, monitoring of expiration date of medications, and reduction of about eight minutes of nursing documentation for each patient [9].

Liang *et al.* also developed an event simulation model to evaluate the clinic’s performance and identify opportunities to enhance workflows and appointment scheduling in the chemotherapy ward. The findings showed that factors influencing clinic scheduling were the number of patients, the interval between arrival time and appointment time, number of patients requiring testing, the appointment scheduling method, the number of nurses, and the number of chemotherapy beds [10]. Francis used the workflow management technology in the Elekta Oncology Information System to provide the possibility of customizing workflows, reviewing work duties, providing care plans, and evaluating activities using quality checklists [11].

Ando developed an oncology information system at the National Institute of Radiation Sciences of Japan, which emphasized the Integrated Healthcare Enterprise (IHE) Data Standard to describe workflows in the oncology information system [12]. Another study by Evans *et al.* in Canada showed that a review of business processes for the optimal use of the oncology information system, selection of appropriate technologies, and definition of new workflows should be considered [13]. Also, in another study, the implementation of a planned workflow in the oncology information system led physicians to devote more time to patients and care for them, and the development of the system in Japanese made it easier for users to communicate with the system [14].

Since the quality of care for cancer patients is affected by the availability of accurate data, and existing hospital information systems do not effectively meet the needs of cancer professionals by providing large volumes of diverse and sporadic data [15], the oncology information system is needed to enhance the access, organization, and management of cancer-related data. Also, having a thorough understanding of the workflows and processes of caring for cancer patients before designing the oncology information system will lead to improved documentation and increased the cost-effectiveness of care services. The purpose of this study was to identify workflows related to cancer care.

## Material and Methods

This single-step study was conducted in 2019 using a qualitative narrative method. To this end, in-depth semi-structured interviews were conducted to gather participants’ points of view. The data were collected from the oncological wards of five educational and therapeutic centers, including Imam Khomeini Medical Center affiliated to Tehran University of Medical Sciences, Shohadaye Tajrish and Taleghani Medical Center affiliated to Shahid Beheshti University of Medical Sciences, and Hazrat Rasoul Akram and Ali Asghar Pediatric Medical Center affiliated to Iran University of Medical Sciences. The participants were clinical and nonclinical staff working at six university hospitals equipped with oncology wards, and the participants were engaged in caring for cancer patients and recording their data (N = 32). Clinical staffs included oncologists, radiologists, pathologists, and nurse heads, and non-clinicians were health information management officials. Before conducting the interviews, the interview guide containing 11 general questions was developed based on the literature review. The validity of the instrument was confirmed by experts and professors, and its reliability was confirmed by a test-retest method. Interviews were recorded using two audio recorders, and in cases where interviewers were not allowed to record audio, the interviews were carefully transcribed. The interviews were then carefully transcribed, and each interview was repeated and listened to several times for greater accuracy. A five-step framework analysis process was used to analyze the data collected through the interviews, including familiarization, identifying a thematic framework, indexing, charting, and mapping and interpreting. Framework analysis is a method used in applied research to obtain information and provide specific recommendations [16, 17]. Finally, by comparing the observed relationships, concepts, contradictions, and theories, the themes were extracted using MAX QDA software (version 12).

## Results

In the first phase, one of the selected hospitals declined the interview invitation, and eventually, 27 were ready to participate in the interview. In this study, of 27 eligible people, 25 participated in the study. The interviews lasted an average of 40 minutes. The participants’ demographic data are presented in Table 1.

**Table 1:** The participants’ demographic data.

Variable		Frequency (%)
Sex	Male	8 (32%)
	Female	17 (68%)
Age	39-30	5 (20%)
	49-40	11 (44%)
	59-50	9 (36%)
Education	Bachelor’s	12 (48%)
	Master’s	3 (12%)
	Specialist	3 (12%)
	Subspecialist	7 (28%)
Occupation	Blood oncology	4 (16%)
	Cancer radiation therapy	3 (12%)
	Pathologist	3 (12%)
	Head nurse	10 (40%)
	Health information manager	5 (20%)
Cancer service records	1-5	3 (12%)
	6-10	4 (16%)
	11-15	8 (32%)
	16 years and more	10 (40%)

As shown in Table 1, most of the participants were females (n = 17, 68%), and the participants in the age group of 40-49 had the highest frequency (n = 11, 44%). Also, the participants with a bachelor’s degree had the highest frequency (n =12, 48%). Furthermore, those who had a working experience of 16 years or more in the field of cancer had the highest frequency (n =10) compared to other groups.

**Table 2:** Themes, categories, and subcategories related to cancer patient care workflows.

Themes	Categories	Subcategories
Cancer patient care workflows	Cancer diagnosis workflows	The patient’s referral to the clinic to check his/her conditions
		The classification of cancer diagnosis workflows
		Workflows in the pathology ward
	Cancer treatment workflows	Types of treatments offered to cancer patients
		Workflows in the chemotherapy ward
		Workflows in the radiotherapy ward
		The classification of workflows for treating various types of cancer

The findings of the study indicated the main themes of cancer patient care workflows could be summarized in two categories, including cancer diagnosis workflows and cancer treatment workflows. The related categories and subcategories are shown in Table 2.

### Category 1: Cancer diagnosis workflows

The cancer diagnostic workflows are divided into three subcategories, including the classification of workflows to diagnose different kinds of cancer, the patient visits to the clinic to have his/her conditions examined, and the workflows in the pathology ward, as presented below.

#### The classification of cancer diagnosis workflows

Most interviewees believed that it was not possible to classify workflows for diagnosing the types of cancers. To diagnose colon cancer, colonoscopy, and the review of the patient’s conditions are required. Besides, gastric cancer can be diagnosed by endoscopy and the review of the patient’s conditions, while lung cancer can be detected through radiology and computed tomography (CT) scans. On the other hand, head and neck cancers are diagnosable by physical examination and biopsy, and prostate cancer can be diagnosed by the prostate-specific antigen test. Blood cancer is typically detected by bone marrow and lymph node biopsy, and breast cancer is diagnosed by breast mammography. Besides, cervical cancer can be checked and detected through the pap smear test. Generally speaking, the definitive diagnosis of cancer is possible by biopsy and performing pathological examinations. One radiologist stated:

“It is not possible to use a general procedure for diagnosing cancers because, for example, the role of CT scan, endoscopy, colonoscopy, and endosonography is important in gastrointestinal tumors. In soft-tissue tumors, magnetic resonance imaging (MRI) plays a very prominent role. MRI is also important in brain tumors. Ultrasound and mammography are important in breast tumors. MRI and ultrasound are important in detecting prostate cancer. In women’s tumors, surgery is usually the main priority. So the categorization becomes too general and not valuable. Because each tumor mass can be detected differently, and the ones I mentioned were the most commonly used procedures”.

#### The patient’s referral to the clinic to check his/her conditions

The interviewees stated that the patient first refers to the clinic to have their illness diagnosed. There, the physician checking the patient’s condition and confirming cancer orders that the patient must be hospitalized. The patient is then admitted to the relevant ward and makes an appointment for hospitalization and is visited on a regular basis by a physician or resident. The data needed to check the patient’s condition include the patient’s checkup records such as clinical examinations, symptom assessment, family history, risk factors, and data from paraclinical tests such as ultrasound, CT scans, MRI, and medical and pathological examinations. One radiotherapist stated:

“The definitive diagnosis of cancer is possible through pathological examinations. Some patients undergo endoscopy first, and biopsies are taken from the mass, as in gastrointestinal cancers. Some have accidentally taken images of the lungs or have some symptoms and have images taken, and then they realized that they had a mass and underwent biopsy. Some also refer to the clinic and state that they have a mass, such as a testicle or a breast mass”.

#### Workflows in the pathology ward

According to pathologists, the procedure for acceptance of the sample until the final diagnosis is as follows. First, the sample is taken in the operating room, then it is recorded in the biopsy laboratory, and the physician’s request is entered into the hospital information system. Next, the sample is taken to the pathology room and cut. Finally, the sample is examined under the microscope, and the final diagnosis is made.

The interviewees believed that there are different workflows in the pathology ward for cancer diagnosis. Some cancers are diagnosed with normal tissue staining. For example, lymph node cancer may be detected with normal staining under a microscope without additional work. But the vast majority of tumors removed from the body require some additional work, such as cytometry with a flow cytometer, staining of biological and tissue samples to quantitatively and qualitatively measure the number of protein antigens, and make a definitive diagnosis. In this regard, another pathologist stated:

“Given that some specimens are malignant tumors and require more specialized tests, they are resected in the immunohistochemistry ward and examined with different markers. Eventually, the kind of tumor, its degree, and the chemotherapy drug affecting it are identified”.

### Category 2: Cancer treatment workflows

The cancer treatment workflows are divided into three subcategories, including the types of treatments offered to cancer patients, the workflows in the chemotherapy ward, and the workflows in the radiotherapy ward, as discussed below.

#### Types of treatments offered to cancer patients

The interviewees stated that a variety of treatments offered to cancer patients are surgical treatment, radiotherapy, or comprehensive treatment, including chemotherapy, hormone therapy, and new drugs. Also, these treatments can be combined. One oncologist stated:

“Surgery, chemotherapy, and radiotherapy are used in most advanced cancers. In less advanced cancers, one or two of these may be used. The classification of these cases depends on the stage of cancer”.

#### Workflows in the chemotherapy ward

Most interviewees reported that cancer patients are referred to a radiologist or oncologist for chemotherapy. Chemotherapy is a type of cancer treatment in which drugs are used to destroy cancer cells. Depending on the type of cancer and its progression, chemotherapy can treat or control cancer and can improve its symptoms. The choice of medication by the physician also depends on factors such as the type of cancer the patient is suffering from, past chemotherapy, and whether the patient has other problems and diseases such as diabetes or heart problems, and so on. If necessary, the patient’s paraclinical information is completed based on a CT scan or MRI results, and then a chemotherapy protocol is planned. Then, the required tests are requested for that protocol to determine if the patient can tolerate the treatment protocol. The drugs are then administered to the patient in a hospital or outpatient setting. According to the interviewees, the chemotherapy protocol includes medication names and sequences, the number of chemotherapy courses, the interval between courses, and how to take medications (intravenously, subcutaneously, or orally). One radiotherapist said:

“Chemotherapy programs are very diverse and different from one another. The length and duration of chemotherapy depend on factors such as the type of cancer and its progression, the purpose of treatment (to treat, control or reduce the symptoms of cancer), the type of chemotherapy, and the patient’s body’s response to chemotherapy”.

#### Workflows in the radiotherapy ward

According to interviewees, the patient is referred to a radiotherapy specialist after the cancer is confirmed. The treatment plan is then set up by a radiologist. The treatment plan may include radiotherapy along with other complementary therapies such as chemotherapy, hormone therapy before or after radiotherapy. Before radiotherapy, the patient meets his/her physician. During these appointments, the patient is examined, his/her conditions are checked, and sometimes imaging is performed. The physician may tell the patient about the benefits of radiotherapy, its side effects, and what to do during and after treatment. The patient can then choose whether to undergo treatment or not.

If the patient agrees to undergo radiation therapy, the radiologist determines whether radiotherapy is performed externally or internally. The simulation CT scan is then performed at one of the radiology centers in coordination with the medical center, and after receiving the CT scan results, the radiologist specifies the exact location of the tumor (a process that is called contouring). The results are referred to a medical physicist for treatment planning and ultimately reconfirmed by a radiologist. In the first session, a radiologist meets the patients to ensure his/her confirmation, and this will be done by the radiotherapist in other sessions. After radiotherapy is completed, the person should be monitored for the rest of their lives. Follow-up measures include examinations and checkups performed by a radiologist to examine the effects of radiotherapy, the recurrence of cancer symptoms, and the late complications of cancer, and talks to the patient about the treatment and care that should be taken.

The detailed design of a treatment plan plays an important role in the patient’s successful treatment. The treatment plan is designed in three stages:

• Design. At this step, the malignant tumor must be identified in such a way as to measure its size and position in the body accurately. The information needed for this step can be obtained through conventional imaging, MRI, positron emission imaging, CT scans, and ultrasound;
• Simulation. After calculating the amount of radiation to be received by the tumor, the patient is transferred to a simulator to determine the patient’s position in front of the accelerator. The simulator and images are used to make sure the tumor is in the right position and receive the required therapeutic dose;
• Treatment. At this stage, the tumor is exposed to gamma rays emitted from a cobalt source or high-energy X-rays produced by a linear accelerator, and the treatment process is performed.

One radiologist said:

“Since radiotherapy usually takes a long time, such as a month or a month and a half, the patient is frequently visited by a cancer radiotherapist during radiotherapy and the radiotherapy problems and changes that need to be made in the radiation therapy process are checked during these frequent visits”.

## Discussion

The oncology information system is used to manage cancer patients’ information in hospitals and monitor different aspects of cancer patient care. Also, by evaluating diagnostic data, defining workflows and clinical protocols, helping to make better decisions using clinical decision support systems, and providing structured treatment plans, the system can improve the care provided to cancer patients and reduce treatment errors [12]. Therefore, in many countries, the oncology information system has been used. This system covers the roles and responsibilities of the clinical staff and facilitates the work of physicians in the treatment of cancer patients through quality assurance and evaluation programs [13].

Our findings showed that cancer patient care workflows were divided into cancer diagnosis and treatment workflows. Cancer diagnosis workflows were categorized into diagnostic workflows, patient’s referrals to the clinic, and workflows in the pathology ward. Besides, cancer treatment workflows include the types of treatments offered to cancer patients, workflows, chemotherapy and radiotherapy wards. The importance of workflows related to the diagnosis and treatment of cancer has been highlighted in numerous studies. For example, Yang created a mockup to predict the workflows in the radiation therapy ward and implement them on a tablet. The users’ interview results showed a high willingness of the radiotherapy staff to change their current workflows. The users suggested the use of electronic protocols as a good way to support their workflows [2]. Wagner *et al.* also conducted a study to define a single clinical documentation workflow for a variety of cancers. Finally, the implementation of this electronic documenting technique was evaluated in five clinical centers, which was desirable based on the results [8]. Donald also used the organizational development model to reconstruct radiotherapy workflows in an outpatient cancer center. After collecting quantitative and qualitative data from stakeholders, he presented a functional change plan that ultimately showed a reduction in time in the radiotherapy process [18].

Liu and Wen also created a chemotherapy care information system that identified key workflows and processes in the chemotherapy ward, which ultimately led to evidence that this system reduced drug errors, helped to monitor expiry dates and reduced nursing documentation time. User satisfaction with the system was also very high [9]. Lianga also developed an event simulation model to enhance workflows and appointment scheduling at the cancer clinic. The evaluation results indicated a reduction in waiting time, a reduction in the workload of the clinic, and an optimal allocation of resources [10]. In another study, Pirnejad explored the impact of using a protocol-based information system on chemotherapy processes. The four workflows related to chemotherapy, including the period of a chemotherapy course, the delay in its implementation, the timing of documentation, and the patient’s satisfaction, were assessed. Finally, the findings showed that using the protocol-based information system by eliminating unnecessary and repetitive tasks reduces the execution time and increases efficiency in workflows [19]. Therefore, it seems that considering cancer care workflows to design an oncology information system is one of the factors affecting the success of the system. Besides, since the use of these systems can improve the quality of care, improve documentation, and optimize resource allocation, taking into account the performance of these systems and adapting them to workflows seems necessary.

One of the limitations of the present was the lack of cooperation of some experts and their inaccessibility. Thus, the researcher tried to provide further explanations about the significance of the study and communicate with them.

## Conclusion

The availability of reliable, up-to-date, and cancer-related data is essential to facilitate screening, prevention, diagnosis, treatment, mental health care, and pain relief for cancer patients. In addition to providing such data, oncology information systems in other countries have played an important role in planning, managing services, monitoring cancer, and facilitating research. Therefore, since the use of oncology information systems improves the quality of care, improves documentation, optimizes the allocation of resources, and increases the cost-effectiveness of care services, this study aimed to identify cancer workflows related to the design of the oncology information system. It is hoped that the results of this study will provide a suitable framework for designing a conceptual model of the oncology information system and lead to enhanced quality of services provided to cancer patients.

## Acknowledgments

This study was supported by the Iran University of Medical Sciences and was conducted under the code: IUMS/SHMIS-94/36.

## Conflict of Interest

The authors declare that there is no conflict of interest.
